# Supplementary material for: A norovirus gastroenteritis outbreak in an Australian child-care center: A household-level analysis
Source: PLoS One. 2021 Nov 2;16(11):e0259145. doi: 10.1371/journal.pone.0259145 (PMC8562815; doi:10.1371/journal.pone.0259145)
Supplement: Attachment — Submitted filename: rebuttal letter v03.docx [file pone.0259145.s002.docx]

1. **“The two generation model”/” The two generation network model” is not a standard model. I suggest the authors to remove the term in the main text and Figure 2 caption but describe the use of infector/infectee pairs (two generation) for the analysis as is (as have already done).**
   a. We have removed the three references to the term “*two generation model*”, and we agree with this recommendation.
